# Vitamin D supplementation improves well-being in patients with frequent respiratory tract infections: a post hoc analysis of a randomized, placebo-controlled trial

**DOI:** 10.1186/s13104-015-1504-2

**Published:** 2015-09-29

**Authors:** Peter Bergman, Anna-Carin Norlin, Susanne Hansen, Linda Björkhem-Bergman

**Affiliations:** Division of Clinical Microbiology, Department of Laboratory Medicine, Karolinska Institutet, Karolinska University Hospital, Huddinge, 141 86 Stockholm, Sweden; Division of Clincal Immunology, Karolinska Institutet, Karolinska University Hospital Huddinge, 141 86 Stockholm, Sweden; Infectious Disease Clinic, Karolinska University Hospital, 141 86 Stockholm, Sweden

**Keywords:** Vitamin D, Immunodeficiency, Well-being, Quality of life, Supplementation, Clinical trial, Placebo

## Abstract

**Background:**

The aim of this study was to test the hypothesis that vitamin D supplementation improves well-being in patients with frequent respiratory tract infections (RTIs). We performed a post hoc analysis of a randomized, placebo-controlled and double-blind study in which patients with frequent RTIs were randomized to placebo or vitamin D (4000 IE/day for 1 year, n = 124). At the last visit of the study, patients were asked to perform a general assessment of their well-being during the study.

**Results:**

The majority of patients, both placebo- and vitamin D treated, stated that they had felt ‘better’ during the study; 52 % in the placebo group and 70 % in the vitamin D group, relative risk 1.3 (95 % CI 1.0–1.8; p = 0.06, Fisher’s exact test). Statement of better well-being was associated with an increase in 25-hydroxyvitamin D (25-OHD) levels (p < 0.001). In contrast, worse well-being was associated with unchanged 25-OHD levels. Notably, a 25-OHD level above 100 nmol/L at the study end was associated with a higher chance of having a better well-being (p < 0.01). Four patients on anti-depressive treatment could terminate their antidepressant medication during the study. These patients had a significant increase in 25-OHD levels from low levels at study-start.

**Conclusion:**

Vitamin D supplementation to patients with frequent RTIs might be beneficial, not only for infections, but also for their general well-being. However, given the post hoc design of this study, these findings need to be confirmed in additional clinical trials before firm conclusions can be drawn.

Trial registration: http://www.clinicaltrials.gov (NCT01131858), registered March 22, 2010

## Background

Patients with primary immunodeficiency often have an impaired quality of life and a high risk for depressive and anxiety disorders [1]. In a longitudinal study of 92 patients with common variable immunodeficiency (CVID), an assessment of general health showed lower scores in these patients compared to patients with other chronic diseases. Approximately one-third of all patients in the cohort were at risk of anxiety/depression, and two-thirds of the females [1].

Vitamin D is important for a healthy immune system and strengthens innate immunity by inducing synthesis of antimicrobial peptides [2]. In addition, vitamin D has broad anti-inflammatory effects on the adaptive immune system [3, 4]. Low levels of 25-hydroxyvitamin D (25-OHD) are associated with an increased risk of respiratory tract infections (RTIs) [5]. Based on these findings, we conducted a randomized, placebo controlled and double blind study where patients with frequent RTIs were randomized to placebo or vitamin D (4000 IE/day for 1 year). In this study (n = 124) we could show that vitamin D treated patients (n = 62) had significantly reduced infectious symptoms, measured as “infectious score”, and a 60 % reduction of antibiotic consumption compared to the placebo group (n = 62) [6].

Interestingly, vitamin D also seems to play a role in the central nervous system. Several reports have shown that low vitamin D levels is associated with increased risk of pain [7, 8] and that vitamin D supplementation can reduce depressive symptoms [9]. Thus, we hypothesized that vitamin D supplementation could improve the overall well-being in patients with frequent RTIs. To test this hypothesis, we performed a post hoc analysis of our previously performed clinical trial [6]. The analysis was based on an assessment of well-being during the study year and this information was related to vitamin D levels and use of antidepressive medication.

## Methods

### Study cohort

We performed a post hoc analysis of data from our previously performed clinical trial [6]. This was a prospective, randomized and double-blind placebo-controlled study of vitamin D_3_ supplementation (4000 IU/day) in patients with an increased susceptibility to respiratory tract infections. The double-blind design implies that the randomization to placebo or vitamin D was blinded for both patients and the study-physicians and study-nurses. Patient characteristics and inclusion criteria are described in details in the original article [6]. The current post hoc analyses is based on the per protocol cohort, which completed the study (n = 124). Of these, n = 62 had placebo-treatment and 62 vitamin D-treatment; thirty-three were men and 91 women. The raw data is available from the corresponding author upon request.

In the original study we asked the patients at the end of the study (last visit) about their overall “well-being” during the study period. The overall health during the study year was assessed by a global assessment approach by asking a single question: “How do you rate your overall health during the study year compared with the year before inclusion”. The questionnaire contained the predefined answers: 1 = better than before, 2 = worse than before, 3 = no change or 4 = other.

**Fig. 1:**
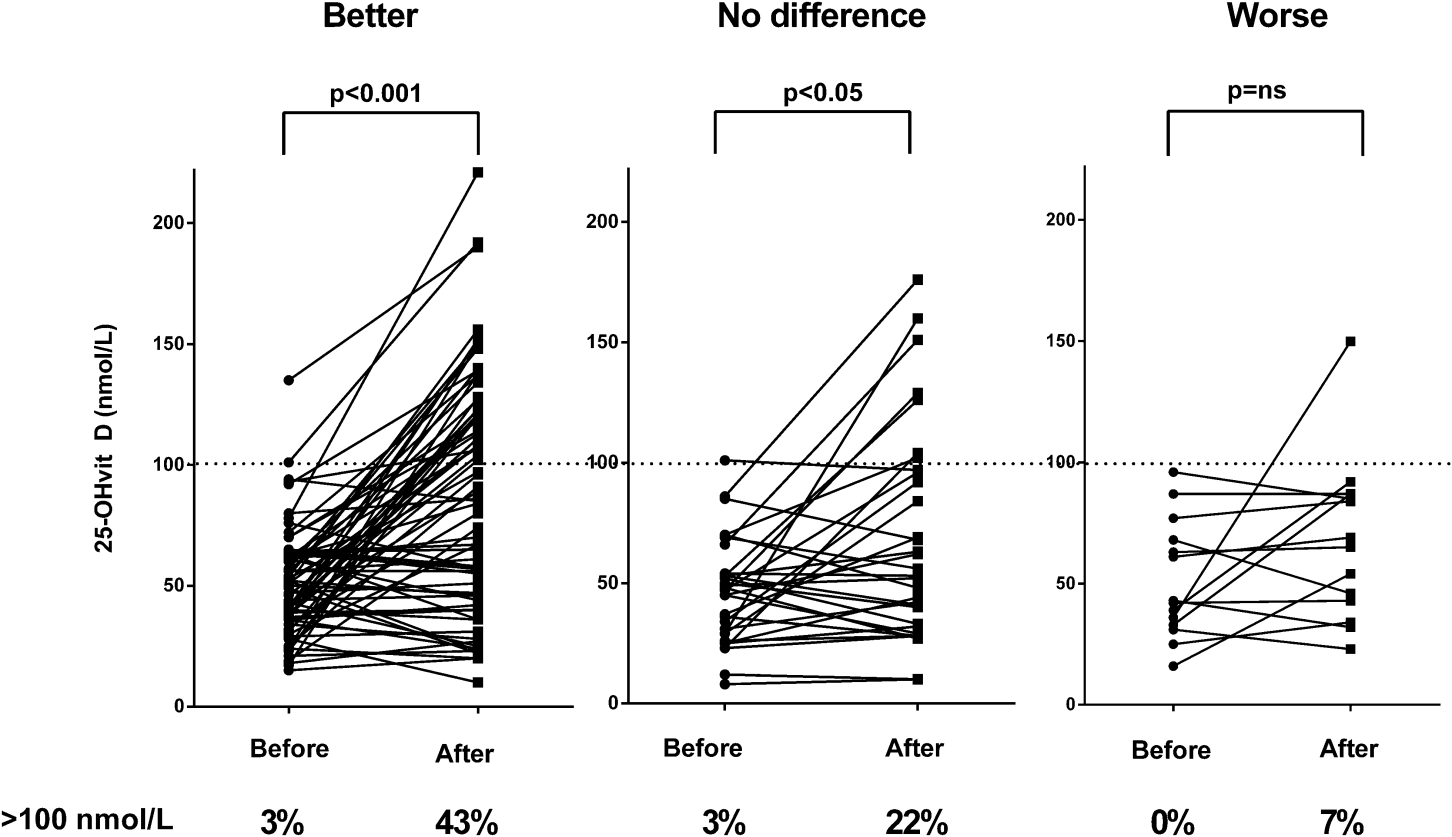
Well-being in patients with immunodeficiency disorders before and after 1 year of supplementation with vitamin D. In patients with a better well-being during the study (n = 72) there was an average increase in 25-hydroxyvitamin D levels (25-OHvitD) (p < 0.001, Student’s t-test). Likewise, in patients with no difference in well being (n = 32) there was an average increase, but less pronounced (p < 0.05, Student’s t-test). In patients stating a worse well-being (n = 14) there was no significant increase (*ns* not significant)

**Fig. 2:**
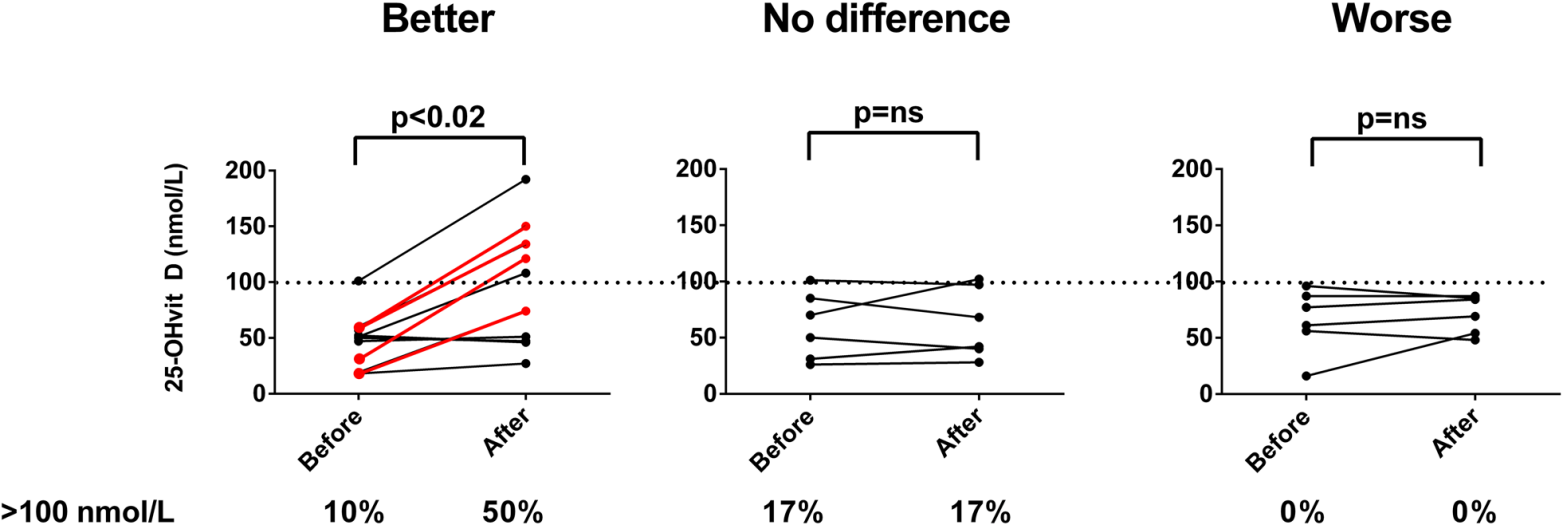
Well-being in patients with immunodeficiency disorders taking antidepressants, before and after a 1 year vitamin D study. In patients with a better well-being during the study (n = 10) there was an average increase in 25-hydroxyvitamin D (25-OHvitD) levels (p < 0.001, Wilcoxon signed rank test). Four patients could terminate their antidepressant therapy during the study (*red lines*). In patients stating no difference (n = 6) or worse well-being (n = 6) there was no change in average 25-OHvitD levels (p = not significant)

All statistical tests were performed using GraphPad Prism v. 6.00 and p < 0.05 were considered statistically significant. The data including the whole cohort (Fig. 1) showed Gaussian distribution and thus mean values and SD are presented as well as 95 % confidence interval (CI). Student’s t-test was used to study the difference between before and after treatment. In the cohort of patients on anti-depressants (Fig. 2) the data lacked Gaussian distribution and thus non-parametric Wilcoxon signed rank test was used.

### Ethical statement

The study was approved by the regional Ethical Review Board at Karolinska Institutet, Stockholm, Sweden and by the Swedish Medical Products Agency. The study was registered at http://www.clinicaltrials.gov (NCT01131858) prior to inclusion of the first patient (registered March 22, 2010, approved May 26, 2010) and was conducted in accordance with the declaration of Helsinki. Written informed consent was obtained from all participants prior to inclusion.

## Results

### Assessment of well-being during study

The majority of patients, both placebo- and vitamin D treated, stated that they had felt ‘better’ during the study; 52 % in the placebo group and 70 % in the vitamin D group, relative risk 1.3 (95 % CI 1.0–1.8; p = 0.06, Fisher’s exact test). Among the patients that stated a better well-being during the study (n = 72) there was a significant increase in 25-hydroxyvitamin D levels (25-OHD levels) during the study, mean levels increased from 50 (SD ±22) to 87 (SD ±48) nmol/L (p < 0.001); 95 % CI before 45–55; after 76–99 nmol/L. Among patients who reported no difference in well-being (n = 32) there was also an increase in 25-OHD levels, but to a lesser extent; mean levels increased from 46 (SD ±22) to 68 (SD ±44) nmol/L (p < 0.05); 95 % CI before 38–54; after 52–84 nmol/L. Among patients who stated a worse well-being during the study (n = 14) there was no significant increase in 25-OHD levels, mean levels increased from 51 (SD ±24) to 68 (SD ±33) nmol/L (p = 0.14); 95 % CI before 37–54; after 49–87 nmol/L. A level of 25-OHD above 100 nmol/L seemed to be associated with a higher chance of experiencing a better well-being (p < 0.01, χ^2^ test, Fig. 1). Levels above 75 nmol/L were not significantly associated with a better well-being (p = 0.16).

### Use of antidepressive drugs in the study

Data about anti-depressive medications were recorded in the original study [6]. At the study start 22 patients were taking antidepressants, 2 men and 20 women. The mean 25-OHD level was 56 nmol/L among these patients (95 % CI 45–68 nmol/L) which was mainly the same as in the whole cohort; mean 49 nmol/L (95 % CI 45–53 nmol/L). At the end of the study, 10 of these 22 patients stated a better well-being, 6 answered no difference and 6 reported a worse well-being (Fig. 2). The patients that had a better well-being during the study had a mean significant increase in 25-OHD levels (p < 0.001), whereas the other patients had no increase (Fig. 2). Interestingly, four subjects could terminate their anti-depressive treatment during the study and all these individuals reported a better well-being (marked with red lines in Fig. 2). All these subjects had a significant increase in 25OHD-levels, from low levels at study-start, 19–57 to 74–150 nmol/L at the study-end.

## Discussion

We and others have shown that vitamin D supplementation may be beneficial for preventing RTIs (reviewed in [10]). Here, we show that a significant increase in 25-OHD levels is associated with a better well-being in patients with frequent RTIs. Based on these data, 100 nmol/L appears to be a tentative threshold for a beneficial effect on well-being in these patients. Interestingly, when 100 nmol/L was tested as a threshold for infectious score, a similar beneficial effect could not be observed (data not shown). Thus, the reporting of a better well-being could not solely be explained by a reduced infectious burden, suggesting that other mechanism could have contributed to the result.

In fact, vitamin D supplementation may be beneficial in the treatment of depression but the effects seems to be moderate [11] and the results are still inconclusive [9]. In addition, several studies have shown that vitamin D supplementation may reduce chronic pain [7, 8, 11, 12]. In a randomized, placebo-controlled trial in elderly (n = 120) vitamin D in a single megadose increased quality of life and reduced pain [13]. To our knowledge, the present study is the first where vitamin D status in patients with frequent RTIs is correlated to an overall assessment of well-being. An interesting notion is that 52 % of the placebo-treated patients stated a ‘better’ well-being during the study, indicating an important beneficial placebo-effect of “any” treatment (vitamin D or placebo). Usually, the placebo effect is estimated to 30 % of the effect of a treatment but this figure might be underestimated [14].

The present study is carried out in Sweden with large seasonal variability of 25-OHD levels in the population between summer and winter due to limited sunlight exposure during the winter time [7]. The lowest levels of 25-OHD levels in a Scandinavian population are in February–March [15]. In Sweden and Finland depressions are most common in spring-time and the suicide peak is in May [16]. Although many factors might contribute to this fact it could be speculated that low 25-OHD levels during early spring-time might contribute to impaired well-being in the population during spring.

It could not be excluded that the results presented here might at least in part be explained by confounding factors. Fewer respiratory tract infections in patients with a large increase in 25-OHD levels might have contributed to the results of better well-being. Moreover, sufficient 25-OHD levels improves general health [17], thus improved well-being might be explained by better physical condition. Furthermore, a recent article suggested that UVB-light has strong analgesic effects by induction of endogenous opioid-like substances (endorphins) in the skin [18]. Thus, vitamin D levels could only serve as a maker for UVB-exposition and the association between vitamin D levels and well-being could be explained by exposure to the sun and endogenous synthesis of endorphins. However, our study has an interventional design and thus the most reasonable cause of the observed increase in 25-OHD levels is vitamin D supplementation and not increased UVB-exposition, making this explanation less likely.

One limitation of the present study was that the trial was not designed to study well-being and the data presented here is a post hoc analysis. We did not use a validated questionnaire for evaluation of “quality of life” or depression, such as GHQ-12, SF-36 [1] or MADRS [19]. Instead, the overall health during the study year was assessed by a global assessment approach by asking a single question. It should also be noted that the current analysis is performed per protocol and not as intention to treat. However, we believe that the per protocol analysis represent the data in a valid manner, since there was an equal amount of patients excluded from each arm. The reasons for exclusion were mainly due to lack of compliance to the protocol, i.e. not taking the drug or not filling out the diary. Importantly, there was no tendency that any reason for exclusion was associated with a specific allocation (vitamin D/placebo), thus minimizing the risk of bias in a per protocol analysis. In addition, the allocation between vitamin D and placebo does not play a major role in this post hoc analysis, since the most interesting result is based on the measured 25-OHD levels and the relation to well-being and not based on allocation to vitamin D or placebo treatment.

Notably, in the original study vitamin D treatment was well-tolerated and no severe adverse events were reported in the study. Indeed, there were more adverse events reported among the placebo-treated patients than in the vitamin D treated patients, indicating that this is a safe treatment [6].

## Conclusion

Vitamin D supplementation could be a safe, cheap and accessible option to improve both physical health and well-being in patients with frequent RTIs. However, given the post hoc design of this small hypothesis-generating study, these findings need to be confirmed in additional clinical trials before firm conclusions can be drawn.

## Abbreviations

CI: confidence interval
CVID: common variable immuno deficiency
RTI: respiratory tract infection
25OHD: 25-hydroxyvitamin D
GHQ12: General Health Questionnaire
SF36: Short Form 36 Health Survey
MADRS: Montgomery Åsberg Depression Rating Scale
